# Isoprostane F_2α_-VI, a new marker of oxidative stress, increases following light damage to the mouse retina

**Published:** 2007-02-07

**Authors:** Tzvete Dentchev, Yuemang Yao, Domenico Praticó, Joshua L. Dunaief

**Affiliations:** 1 F. M. Kirby Center for Molecular Ophthalmology, Scheie Eye Institute, Philadelphia, PA; 2 Department of Pharmacology, University of Pennsylvania, Philadelphia, PA

## Abstract

**Purpose:**

A number of studies have suggested that retinal light damage involves oxidative stress. These include demonstration of protection by antioxidants, immunohistochemical detection of oxidative stress markers, and upregulation of antioxidant enzymes. Recently a new specific marker of lipid peroxidation (LPO), isoprostane F_2α_-VI, has been developed. This prostaglandin isomer is produced by nonenzymatic oxidation of membrane-linked arachidonic acid. Because it provides an unusually stable and specific measure of LPO, we sought to determine whether its levels would increase following retinal light damage.

**Methods:**

Balb/c mice were exposed to bright fluorescent light for 7 h. Twenty-eight h after light exposure, photoreceptor death was assessed by terminal deoxynucleotidyl transferase-mediated dUTP nick-end labeling (TUNEL) analysis. Isoprostane F_2α_-VI was quantified in retinal extracts by gas chromatography/mass spectrometry. Retinal isoprostane was localized by immunofluorescence.

**Results:**

TUNEL analysis demonstrated photoreceptor cell death after light exposure. Compared with controls, retina extracts from mice exposed to fluorescent light had a significant increase in isoprostane F_2α_-VI levels following light damage. Immunohistochemistry confirmed an increase in retinal isoprostane.

**Conclusions:**

Elevated levels of isoprostane F_2α_-VI, a stable, highly specific marker of lipid peroxidation, confirm earlier reports of light-mediated retinal lipid peroxidation, potentially an important mechanism of retinal degeneration. Further, since levels of isoprostane F_2α_-VI are readily quantified, its measurement provides a new means to specifically monitor retinal oxidative damage caused by prooxidants such as light.

## Introduction

Retinal oxidative stress caused by light exposure has been implicated in the pathogenesis of age-related macular degeneration (AMD) and other retinal degenerations [1,2]. Photooxidative stress is exacerbated by an imbalance between light-induced reactive oxygen species (ROS) and antioxidants. Light damage in rodents is a well-established model system to study retinal degeneration [3]. In this model and many others, photoreceptor death occurs through apoptosis, as determined by terminal deoxynucleotidyl transferase dUTP nick end-labeling (TUNEL) and gel electrophoresis demonstrating apoptosis-specific DNA laddering [4,5]. Similarly, in our Balb/c mouse light damage model, many photoreceptor nuclei label with TUNEL after a 7 h bright, cool-white fluorescent light exposure [6].

Photooxidative stress has been implicated as a mechanism of retinal light damage. Biochemical studies have detected lipid peroxides (LPO) in light-exposed isolated rat outer segments [7]. Immunohistochemistry has demonstrated labeling for markers of oxidative damage [8]. Several antioxidant genes are upregulated following photic injury, including heme oxygenase [9], thioredoxin [8], glutathione peroxidase [10], ceruloplasmin [11], and metallothionein [6,12]. Further, exogenous antioxidants protect the rodent retina from photic injury [13,14].

Polyunsaturated fatty acids (PUFAs), such as arachidonic acid (AA), are an integral component of neuronal membranes, including photoreceptor outer segments [15,16]. They are esterified to phospholipids from which they can be liberated via phospholipases and subsequently metabolized by three enzymatic oxidative pathways: cyclooxygenase, lipoxygenase, and cytochrome P450 [17]. Recently, a novel oxidative pathway was described in humans as well as in animals. In the presence of ROS, PUFA can undergo non-enzymatic oxidation and form a new class of products, which are isomers of the enzyme-derived prostaglandins and generally called isoprostanes [18]. The most extensively studied isoprostanes are isomers of the prostaglandin F_2α_ (PGF_2α_) and for this reason generally termed F_2_-isoprostanes (F_2_-iPs). Since their original description, because of their chemical stability and the rapid development of sensitive methods for their measurement, data have accumulated supporting the concept that they are the most reliable index of lipid peroxidation in vivo [19]. F_2_-iPs are present in detectable levels in all normal animal and human biological fluids and tissues. We developed an assay for specific F_2_-iPs isomers using gas chromatography/mass spectrometry (GC/MS) and have found that isoprostane F_2α_-VI is one of the most abundant F_2_-iP in humans as well as in animals [20].

We and others have found that measurement of isoprostane F_2α_-VI in the central nervous system (CNS) specifically reflects oxidative stress in animal models as well as in human Alzheimer's disease (AD). In post mortem brains from patients with AD, levels were increased relative to age-matched normal controls [21]. Levels were also increased in the cerebrospinal fluid of patients with AD and Mild Cognitive Impairment (MCI), suggesting that increased LPO is an early component of AD [22]. A similar increase was also observed in the CNS of an animal model of AD-like amyloidosis, i.e., Tg2576.

Since isoprostane F_2α_-VI is a stable, specific marker of in vivo oxidative stress, we chose to test whether it would be useful as a marker of photooxidative stress in vivo in the mouse retina.

## Methods

### Light damage

**Figure 1:**
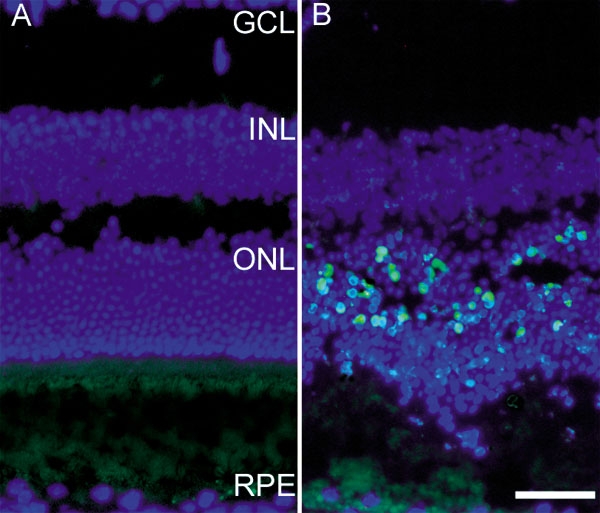
Fluorescence photomicrographs of TUNEL-labeled mouse retinas. Control mice did not have bright light exposure (**A**). Experimental mice were sacrificed 28 h after light exposure (**B**). All nuclei are labeled with DAPI (blue). TUNEL-positive photoreceptors appear blue-green in **B**. Retinal layers are abbreviated as follows: retinal pigment epithelium (RPE), outer nuclear layer containing photoreceptor nuclei (ONL), inner nuclear layer (INL), and ganglion cell layer (GCL). Scale bar indicates 50 μm.

We obtained approval from the University of Pennsylvania Animal Care and Use Committee and followed animal care guidelines comparable to those published by the Institute for Laboratory Animal Research. We then took eight-week-old male Balb/c (albino) mice (Jackson Laboratory, Bar Harbor, ME) with undilated pupils and dark adapted them for 24 h and exposed them for 7 h to 10 000 lux cool white fluorescent light from 2 am to 9 am following guidelines described in reference [6]. This light dose in nonreflective cages causes death of less than half of the photoreceptors. The mice were euthanized 28 h after light damage, a time point at which TUNEL-positive photoreceptors are evident [12] (Figure 1). Retinas from the right eye of each mouse were immediately isolated and collected on dry ice for GC/MS quantification of isoprostane, and the left eye was saved in fixation buffer (see following section) after enucleation for TUNEL analysis. Control group mice were dark adapted and exposed to the normal ambient light of the animal facility (200 lux), then eyes were studied as previously described.

### TUNEL analysis

The enucleated eyes used for the TUNEL analysis were immersed in 4% paraformaldehyde and fixed for 12 h. This fixation facilitates subsequent dissection and generally improves cryosection quality. Eye cups were generated by removing the anterior segment, cryoprotected with 30% sucrose overnight, and then embedded in Tissue-Tek OCT (Sakura Finetek, USA, Torrance, CA). Next 10 μm frozen sections were cut in the sagittal plane through the optic nerve head. The TUNEL in situ apoptosis detection kit (Roche, Mannheim, Germany) was applied to detect cleaved DNA in the frozen sections using the manufacturer's protocol.

### Isoprostane quantification

For biochemical analysis of iPF2α-VI levels, mice (n=16) were euthanized, and their retinas were isolated immediately and collected on dry ice. Retinas were homogenized, and total lipids were extracted with ice-cold Folch solution, chloroform/methanol (2:1, vol/vol). The solution was then vortex-mixed and centrifuged at 800 g for 15 min at 4 °C. Lipid extracts were subjected to base hydrolysis by adding aqueous 15% KOH and then incubated at 45 °C for 1 h for measurement of total isoprostane F_2α_-VI by negative-ion chemical ionization GC/MS as previously described [21,22]. Briefly, a known amount of the internal standard, d_4_-8-12, *iso*-iPF_2α_-VI, was added to each sample. After solid-phase extraction, the samples were derivatized, purified by thin layer chromatography, and analyzed. Quantification was performed using peak area ratios [21,22].

### Immunohistochemistry

Following blocking for 1 h at room temperature in phosphate buffered saline (PBS), 1% bovine serum albumin (BSA), and 1% normal donkey serum, cryosections were exposed to anti-isoprostane F_2α_ antibody (Abcam, Cambridge, UK, dilution 1:500) overnight at 4 °C in PBS, 0.1% triton, and 1% BSA. Control sections were treated identically but with omission of primary antibody. Sections were then washed three times, 5 min per wash in PBS. Secondary antibody (donkey anti-rabbit labeled with Cy-3; Jackson ImmunoResearch Laboratories, Inc., West Grove, PA) was then applied for 1 h at room temperature. Sections were again washed three times for 5 min each in PBS. Nuclei were counterstained with DAPI (1.5 μg/ml)-supplemented Vectashield mounting medium. Epifluorescence microscopy was performed with a Nikon TE-300 microscope (Nikon Inc., Tokyo, Japan) and SpotRT Slider camera (Diagnostic Instruments, Inc., Sterling Heights, MI) with ImagePro Plus software, version 4.1 (Media Cybernetics, Silver Springs, MD).

## Results

It has been shown previously that bright light exposure causes photoreceptor apoptosis [4]. To confirm that our light exposure protocol resulted in photoreceptor death, we performed TUNEL labeling on mouse retinas 28 h after light exposure. As expected, numerous TUNEL-positive photoreceptors were present in retinas from bright light-exposed mice (Figure 1), but not in retinas from mice maintained at low light levels. Some photoreceptors remained TUNEL-negative and survived for at least two weeks (not shown). As a control, TUNEL labeling was performed on a light-damaged retina with omission of the terminal deoxynucleotidyl transferase. This eliminated the TUNEL label, as expected (not shown). Retinas from all light damaged mice tested in this experiment exhibited TUNEL-positive photoreceptors, confirming that each mouse had experienced retinal light damage.

**Figure 2:**
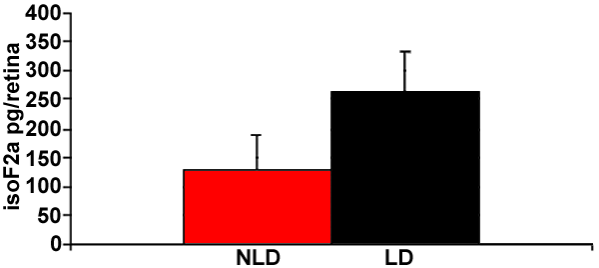
Bar graph showing mean levels of isoprostane F_2α_-VI in mouse retinal extract. The experimental group (LD, n=8 retinas) had been exposed to bright light. The control group (NLD, n=8) was maintained in normal room light conditions. Bars represent standard deviation. p<0.001, Student's two-tailed t-test.

Levels of iPF2α-VI were tested using GC/MS. The sensitivity of GC/MS to detect iPF2α-VI was high enough to find measurable quantities in single mouse retinas. When unexposed retinas (n=8) were compared to bright light-exposed retinas (n=8), the bright light-exposed retinas had a significant increase (p<0.001, Student's two tailed t-test, Figure 2).

**Figure 3:**
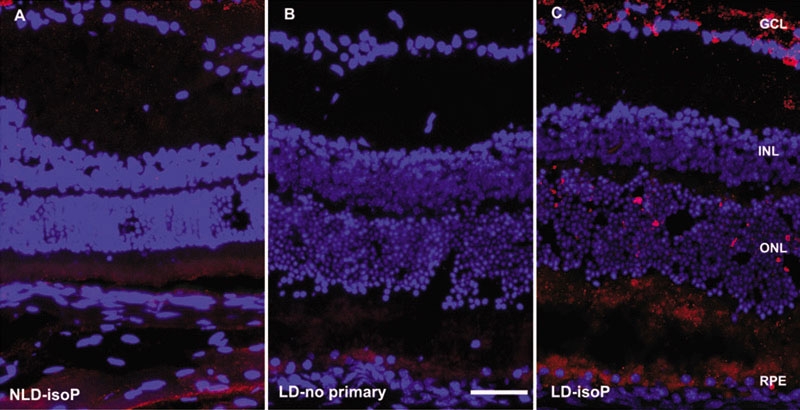
Fluorescence photomicrographs of Balb/c mouse retinas. **A**: Normal retina, no bright light exposure, labeled with anti-isoprostane antibody (red fluorescence). **B**: Control retina without primary antibody after 28 h bright light exposure. **C**: Retina after 28 h bright light labeled with anti-isoprostane antibody exposure. Nuclei are labeled with DAPI (blue). Scale bar indicates 50 μm. Abbreviations are defined as follows: no light damage (NLD), light damage (LD), exposure to anti-isoprostane antibody (isoP).

**Figure 4:**
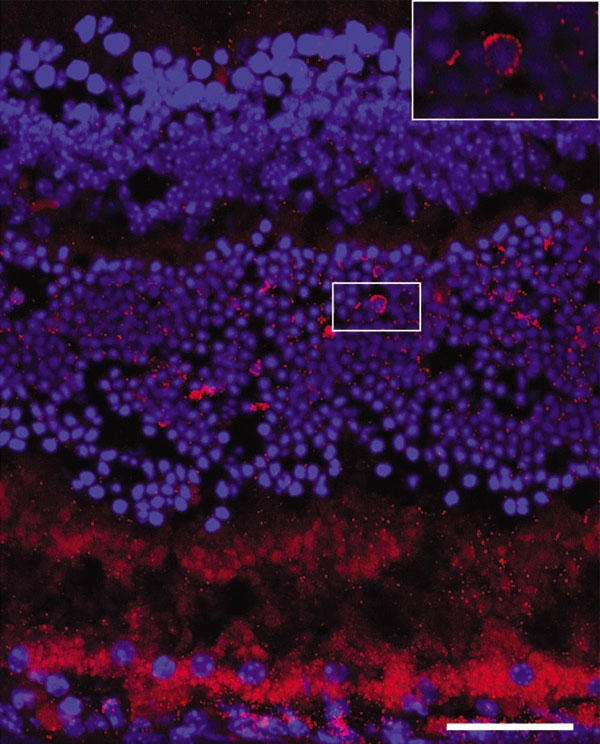
Confocal micrograph of Balb/c mouse retina. Confocal micrograph of Balb/c mouse retina, labeled with anti-isoprostane antibody (red), following 28 h bright light exposure. Nuclei are labeled with DAPI (blue). Inset shows perinuclear label. Scale bar indicates 50 μm.

Immunohistochemistry with an anti-isoprostane F_2α_ antibody (Abcam, Cambridge, UK, dilution 1:500) was performed to determine the location of the isoprostane after light damage. Normal retinas not exposed to bright light had some weak photoreceptor outer segment label (Figure 3A). Retinas from mice exposed to bright light had increased levels of anti-isoprostane immunoreactivity in the outer nuclear layer (ONL) and ganglion cell layer (GCL; Figure 3C). Retinal pigment epithelium (RPE) autofluorescence is visible in the no primary antibody control (Figure 3B). Confocal microscopy (Figure 4) reveals punctate labeling in the ONL, with some cells showing perinuclear labeling (Figure 4, inset). Photoreceptor outer segment labeling is more apparent in this confocal micrograph than in epifluorescence micrographs.

## Discussion

We report herein increased levels of retinal isoprostane F_2α_-VI following bright light exposure. This increase was detected by both GC/MS and immunohistochemistry (IHC). Isoprostane F_2α_-VI quantification represents a new specific and sensitive technique for measuring oxidative stress in the retina and confirms previous reports of increased lipid peroxidation following bright light exposure. Lipid peroxidation is the mechanism by which lipids are attacked by ROS that have sufficient reactivity to abstract an atom of hydrogen from a methylene carbon group in their side chain. The greater the number of double bonds in the lipid molecule, the easier the removal of the hydrogen atom. This explains why PUFA are particularly susceptible to lipid peroxidation. The recent discovery of isoprostane F_2α_-VI, a specific and sensitive marker of lipid peroxidation in vivo, is enabling rapid progress in the measurement of LPO.

During preparation of this manuscript, and after our presentation of this study at ARVO [23], we became aware of a recent publication of similar findings in a well-designed study by another group [24]. GC/MS-based quantification of a different isoprostane, 8 iso-PGF2α, revealed significant elevation in light damaged Balb/c retinas compared to controls. These results were in agreement with our findings. However there are some slight differences: 8 iso-PGF2α- is a class III isoprostane, whereas the isoprostane measured herein, isoprostane F_2α_-VI, is a class VI isoprostane. The advantage of measuring a class VI versus a class III is that while the latter can be formed by both free -radical and enzymatic pathways, the former is formed only by a free-radical mechanism, making it a more specific marker of oxidative stress [19,25]. Further, similar to our study but using a different antibody, IHC conducted by Tanito et al. [24] detected elevated isoprostane levels not just in photoreceptors, but in the inner retina as well.

The elevated levels of isoprostane F_2α_-VI in the inner retina from mice exposed to bright light in our study (Figure 3C) and that of Tanito et al. [24] is somewhat surprising, given that photoreceptors are the cells that degenerate following light damage. This may be due to either isoprostane production in photoreceptors with subsequent release into the extracellular space following phospholipase cleavage or to local production of isoprostane throughout the retina caused by the photooxidative stress. The possibility of photooxidative stress occurring in the inner retina is supported by upregulation of the antioxidant enzyme thioredoxin in the inner retina following light damage [8,26,27]. Omission of the primary antibody resulted in elimination of most of the red fluorescence, ruling out a light damage induced non-specific antibody adherence to the tissue (Figure 3B). The mosaic pattern ONL labeling with the anti-isoprostane F_2α_-VI antibody may result from the relative insensitivity of IHC compared with the more sensitive GC/MS.

Previously retinal oxidative stress and lipid peroxidation has been detected following light damage [7,8]. Isoprostane quantified by GC/MS provides a new specific and quantitative measure of retinal oxidative stress. Thus, the finding of elevated isoprostane in the light damaged retina provides strong evidence that bright light exposure of the retina induces oxidative damage. The role of isoprostane in retinal physiology is unclear, but it is possible that its elevated levels may play a role in retinal cell survival after photooxidative stress.

Future experiments will be aimed toward defining which human and mouse retinal degenerations are associated with elevated isoprostane levels as well as testing the efficacy of protective antioxidants. We will also test a GC/MS assay to measure oxidized docosahexaenoic acid, which is more abundant in photoreceptors than arachidonic acid, and which has been found within drusen in AMD eyes [28].

## References

[1] Taylor HR Munoz B West S Bressler NM Bressler SB Rosenthal FS Visible light and risk of age-related macular degeneration. Trans Am Ophthalmol Soc 1990 88 163 73discussion173-8 2095019 PMC1298584

[2] Cruickshanks KJ, Klein R, Klein BE, Nondahl DM (2001). Sunlight and the 5-year incidence of early age-related maculopathy: the beaver dam eye study.. Arch Ophthalmol.

[3] Noell WK, Walker VS, Kang BS, Berman S (1966). Retinal damage by light in rats.. Invest Ophthalmol.

[4] Hafezi F, Steinbach JP, Marti A, Munz K, Wang ZQ, Wagner EF, Aguzzi A, Reme CE (1997). The absence of c-fos prevents light-induced apoptotic cell death of photoreceptors in retinal degeneration in vivo.. Nat Med.

[5] Portera-Cailliau C, Sung CH, Nathans J, Adler R (1994). Apoptotic photoreceptor cell death in mouse models of retinitis pigmentosa.. Proc Natl Acad Sci USA.

[6] Chen L, Wu W, Dentchev T, Wong R, Dunaief JL (2004). Increased metallothionein in light damaged mouse retinas.. Exp Eye Res.

[7] Anderson RE, Rapp LM, Wiegand RD (1984). Lipid peroxidation and retinal degeneration.. Curr Eye Res.

[8] Tanito M, Masutani H, Nakamura H, Ohira A, Yodoi J (2002). Cytoprotective effect of thioredoxin against retinal photic injury in mice.. Invest Ophthalmol Vis Sci.

[9] Kutty RK, Kutty G, Wiggert B, Chader GJ, Darrow RM, Organisciak DT (1995). Induction of heme oxygenase 1 in the retina by intense visible light: suppression by the antioxidant dimethylthiourea.. Proc Natl Acad Sci USA.

[10] Ohira A, Tanito M, Kaidzu S, Kondo T (2003). Glutathione peroxidase induced in rat retinas to counteract photic injury.. Invest Ophthalmol Vis Sci.

[11] Chen L, Dentchev T, Wong R, Hahn P, Wen R, Bennett J, Dunaief JL (2003). Increased expression of ceruloplasmin in the retina following photic injury.. Mol Vis.

[12] Chen L, Wu W, Dentchev T, Zeng Y, Wang J, Tsui I, Tobias JW, Bennett J, Baldwin D, Dunaief JL (2004). Light damage induced changes in mouse retinal gene expression.. Exp Eye Res.

[13] Li ZY, Tso MO, Wang HM, Organisciak DT (1985). Amelioration of photic injury in rat retina by ascorbic acid: a histopathologic study.. Invest Ophthalmol Vis Sci.

[14] Noell WK, Organisciak DT, Ando H, Braniecki MA, Durlin C (1987). Ascorbate and dietary protective mechanisms in retinal light damage of rats: electrophysiological, histological and DNA measurements.. Prog Clin Biol Res.

[15] Fliesler SJ, Anderson RE (1983). Chemistry and metabolism of lipids in the vertebrate retina.. Prog Lipid Res.

[16] Wetzel MG, Li J, Alvarez RA, Anderson RE, O'Brien PJ (1991). Metabolism of linolenic acid and docosahexaenoic acid in rat retinas and rod outer segments.. Exp Eye Res.

[17] Smith MA, Rottkamp CA, Nunomura A, Raina AK, Perry G (2000). Oxidative stress in Alzheimer's disease.. Biochim Biophys Acta.

[18] Morrow JD, Hill KE, Burk RF, Nammour TM, Badr KF, Roberts LJ (1990). A series of prostaglandin F2-like compounds are produced in vivo in humans by a non-cyclooxygenase, free radical-catalyzed mechanism.. Proc Natl Acad Sci USA.

[19] Pratico D (1999). F(2)-isoprostanes: sensitive and specific non-invasive indices of lipid peroxidation in vivo.. Atherosclerosis.

[20] Pratico D, Lawson JA, Rokach J, FitzGerald GA (2001). The isoprostanes in biology and medicine.. Trends Endocrinol Metab.

[21] Pratico D, MY Lee V, Trojanowski JQ, Rokach J, Fitzgerald GA (1998). Increased F2-isoprostanes in Alzheimer's disease: evidence for enhanced lipid peroxidation in vivo.. FASEB J.

[22] Pratico D, Clark CM, Lee VM, Trojanowski JQ, Rokach J, FitzGerald GA (2000). Increased 8,12-iso-iPF2α-VI in Alzheimer's disease: correlation of a noninvasive index of lipid peroxidation with disease severity.. Ann Neurol.

[23] Dentchev TI, Yao Y, Pratico D, Dunaief JL, Kirby FM. Isoprostane, a new marker of oxidative stress, increases following light damage to the mouse retina. ARVO Annual Meeting; 2004 April 25-29; Fort Lauderdale (FL).

[24] Tanito M, Yoshida Y, Kaidzu S, Ohira A, Niki E (2006). Detection of lipid peroxidation in light-exposed mouse retina assessed by oxidative stress markers, total hydroxyoctadecadienoic acid and 8-iso-prostaglandin F2α.. Neurosci Lett.

[25] Pratico D, Rokach J, Lawson J, FitzGerald GA (2004). F2-isoprostanes as indices of lipid peroxidation in inflammatory diseases.. Chem Phys Lipids.

[26] Tanito M, Masutani H, Nakamura H, Oka S, Ohira A, Yodoi J (2002). Attenuation of retinal photooxidative damage in thioredoxin transgenic mice.. Neurosci Lett.

[27] Tanito M, Nishiyama A, Tanaka T, Masutani H, Nakamura H, Yodoi J, Ohira A (2002). Change of redox status and modulation by thiol replenishment in retinal photooxidative damage.. Invest Ophthalmol Vis Sci.

[28] Crabb JW, Miyagi M, Gu X, Shadrach K, West KA, Sakaguchi H, Kamei M, Hasan A, Yan L, Rayborn ME, Salomon RG, Hollyfield JG (2002). Drusen proteome analysis: an approach to the etiology of age-related macular degeneration.. Proc Natl Acad Sci USA.

